# Resting-state neural dynamics changes in older adults with post-COVID syndrome and the modulatory effect of cognitive training and sex

**DOI:** 10.1007/s11357-024-01324-8

**Published:** 2024-08-29

**Authors:** Boglárka Nagy, Andrea B. Protzner, Balázs Czigler, Zsófia Anna Gaál

**Affiliations:** 1 Institute of Cognitive Neuroscience and Psychology, HUN-REN Research Centre for Natural Sciences, Budapest, Hungary; 2 Department of Psychology, University of Calgary, Calgary, Alberta Canada; 3 Hotchkiss Brain Institute, University of Calgary, Calgary, Alberta Canada; 4 Mathison Centre for Mental Health Research and Education, University of Calgary, Calgary, Alberta Canada; 5 Bajcsy-Zsilinszky Hospital, Budapest, Hungary

**Keywords:** Aging, Post-COVID syndrome, Cognitive training, Resting-state EEG, Multiscale entropy, Spectral power density

## Abstract

Post-COVID syndrome manifests with numerous neurological and cognitive symptoms, the precise origins of which are still not fully understood. As females and older adults are more susceptible to developing this condition, our study aimed to investigate how post-COVID syndrome alters intrinsic brain dynamics in older adults and whether biological sex and cognitive training might modulate these effects, with a specific focus on older females. The participants, aged between 60 and 75 years, were divided into three experimental groups: healthy old female, post-COVID old female and post-COVID old male. They underwent an adaptive task-switching training protocol. We analysed multiscale entropy and spectral power density of resting-state EEG data collected before and after the training to assess neural signal complexity and oscillatory power, respectively. We found no difference between post-COVID females and males before training, indicating that post-COVID similarly affected both sexes. However, cognitive training was effective only in post-COVID females and not in males, by modulating local neural processing capacity. This improvement was further evidenced by comparing healthy and post-COVID females, wherein the latter group showed increased finer timescale entropy (1–30 ms) and higher frequency band power (11–40 Hz) before training, but these differences disappeared following cognitive training. Our results suggest that in older adults with post-COVID syndrome, there is a pronounced shift from more global to local neural processing, potentially contributing to accelerated neural aging in this condition. However, cognitive training seems to offer a promising intervention method for modulating these changes in brain dynamics, especially among females.

**Supplementary Information:**

The online version contains supplementary material available at 10.1007/s11357-024-01324-8.

## Introduction

The global COVID-19 pandemic caused by the severe acute respiratory syndrome coronavirus 2 (SARS-CoV-2) has made lasting changes in our everyday lives and health and socioeconomic systems. There is accumulating evidence that many patients suffer from prolonged and persistent symptoms after remission from the acute COVID-19 infection, including cardiorespiratory, gastrointestinal, endocrine as well as neurological and neuropsychological symptoms. The latter symptoms could present as brain fog, which is characterized by cognitive impairments in attention, concentration, memory, executive function and information processing speed, fatigue, depression, anxiety, myalgia (generalized pain) and sleep problems [1–7]. This chronic state of the COVID-19 infection is called post-COVID syndrome, which lasts for 2 months or more after remission from the acute infection and cannot be explained by other diagnoses [8]. It affects 30–70% of infected patients and may occur regardless of initial illness severity [9–11], but patients who experience severe COVID illness with hospitalization are more likely to develop it [12, 13]. Despite attempts to develop interventions for managing post-COVID syndrome-related symptoms [14, 15], many patients do not recover and experience long-lasting decreased quality of life. Accordingly, a growing number of studies have investigated different biological mechanisms including immune dysfunction (low-grade hyperinflammation [16–18]), vascular dysfunction (endothelial dysfunction [19–21]) and metabolic dysfunction (oxidative stress [22, 23]) underlying post-COVID syndrome to establish a systematic approach in its treatment [24, 25].

Interestingly, there are remarkable sex differences in the course of both acute and chronic phases of COVID-19: while males are more likely to suffer from severe illness during the acute phase [26–29], females are more susceptible to post-COVID syndrome and show a greater symptom load in the chronic phase [30–36]. Heightened chronic risk for females may arise from biological sex-related differences in hormones, stress and immune responses as well as in behaviour and lifestyle [37, 38]. It is therefore important to disaggregate by sex when examining post-COVID syndrome, as well as the possibility of applying distinct therapies in this context. Medicine often prioritizes research on males, as it is easier to test various treatments and drug effects on men without accounting for cyclic hormonal changes. However, we increasingly recognize that this approach may pose risks for women. In this study, we compare females and males to examine potential sex differences, but primarily focus on females, an understudied group, as we investigate the post-COVID syndrome.

Age is another important factor, as it has been greatly emphasized that older age is a risk factor for gaining severe symptoms during both the acute and chronic phases of COVID-19 infection [13, 31, 39, 40]. Previous work also suggests that COVID-19 infection also may accelerate biological aging processes [41]. Chronicity leads to low-grade hyperinflammation, which is associated with accelerated patterns of age-related dysregulation in the immune system [42, 43]. There is also evidence for accelerated epigenetic aging in the chronic phase of COVID-19, as measured with DNA methylation and telomere length [44, 45], and through increased risk for developing neurodegenerative aging disorders like dementia or Alzheimer’s disease [46–51].

Unsurprisingly, there is growing evidence for structural and functional brain abnormalities associated with COVID-19 infection, including widespread white matter disruption [52–54]; hypometabolism mainly in frontal, limbic and olfactory areas [55]; grey matter abnormalities in prefrontal, anterior cingulate, olfactory, limbic and insular regions [56, 57] and alterations in large-scale functional connectivity affecting the default mode, dorsal attention, salience, somatosensory, subcortical and cerebellar networks [58–62]. In this study, we investigate the resting-state neural dynamics in post-COVID syndrome, as measured with EEG. Differences in resting-state neural dynamics have been linked to variations in cognitive function [63–70] and to disorders in brain function such as psychiatric and neurodegenerative disorders [71, 72]. Additionally, these resting-state network dynamics show sex differences: while females show stronger intra-network connectivity, males present more inter-network connectivity [73–75].

Here, we focus on the complexity of the resting-state signal because neural complexity has been connected to adaptive and flexible information processing, stimulus representation and learning and functional integrity [76–81]. It can be measured with different information entropy methods which characterise the unpredictability and regularity of neural activity and its information content. Higher complexity indicates more flexible and stochastic processes in neural network dynamics, while lower complexity shows more rigid, deterministic and regular neural processing [77, 82–84]. A widely used entropy measure on EEG data is multiscale entropy (MSE; [85, 86]) which detects meaningful recurring patterns (signal predictability) at different timescales. This method is sensitive to both linear and non-linear processes, and it can be complemented by more commonly applied spectral power analyses which can only reveal linear patterns [87]. Therefore, entropy measures can be related to the frequency content of the signal in order to investigate neural dynamics [88]. Previous research revealed that local neural information processing is connected to shorter time range MSE and higher frequency-band power, while distributed neural information processing can be detected with longer time range MSE and lower frequency-band power [83, 84, 88–91].

Regarding the aging process, several studies have found increased fine time scale MSE and decreased coarse time scale MSE in older adults compared to younger ones [83, 91–94]. Current results about resting-state frequency band power reveal general age-related differences as well. Higher frequencies (beta) show increased power, while lower frequency bands (delta, theta, alpha) show decreased power during the aging process [91, 94–101]. Together, these findings are interpreted as an age-related shift from more long-range to local information processing flexibility and capacity. This information processing shift predicted better cognitive functions in older adults [102]; however, some studies suggested the importance of both local and more distributed information processing flexibility for better performance in perceptual, attention and lexical tasks in older adults [91, 94]. Additionally, recent work has found direct connections between variations in resting-state neural complexity with differences in behavioural and cognitive performance with aging [103, 104]. Furthermore, patients with dementia, Alzheimer’s or Parkinson’s disease showed less local and more distributed information processing capacity [89, 105–107] as well as generally decreased entropy [108]. Sex differences have not been examined for complexity measures in this context, but studies examining frequency band power show stronger higher frequency band power in females and stronger lower frequency band power in males [109, 110], while Cave and Barry [111] showed larger delta, midline theta, alpha and beta activity in females compared to males. Notably, there has been very sparse and contradictory evidence regarding how the chronic COVID-19 infection may affect neural dynamics: delta frequency band has been reported to both increase [112] and decrease [113] so far.

In this exploratory study, we investigated how brain dynamics are affected by post-COVID syndrome in older adults. We assumed that if cognitive training has a mitigating effect on post-COVID patients, these effects could be detected as general changes in brain function since brain function seems to be more sensitive than behavioural indices and performance in many experimental designs, interventions and treatments due to neuroplasticity and dynamic neural reconfigurations [114–117]. Moreover, it is also important to identify potential methods that could modulate these dynamics in order to support more efficient and adaptable functioning. A promising candidate is cognitive training, where participants regularly practice a specific and demanding cognitive task over weeks or months. The main goal of this type of intervention is not just to improve performance on the trained or similar tasks (near-transfer effect) but to gain more general cognitive and neural benefits (far-transfer effect). Even though there is some debate about the general efficacy of cognitive training in cognitive aging [118–120], there are many existing studies which support the positive effect of it [121–125]. Additionally, it has been shown that cognitive training could support more efficient brain structure and function in older adults by lessening age-associated decreases in cerebral blood flow and white matter integrity, reducing structural shrinkage and compensating for functional dedifferentiation in resting-state networks [126–130]. Adding to this line of research, in our recent study examining the influence of cognitive training in women [131], we found that adaptive cognitive training with a task-switching paradigm could modulate intrinsic neural dynamics in older individuals. More precisely, we also detected the age-related shift from more global to local information processing with non-pathological aging before training; however, this shift lessened with cognitive training in older women as coarse timescale entropy and lower frequency band power (connected to large-scale neural processing) increased from pre- to post-training in the old-training group.

Altogether, previous research suggests that being female and being older confer increased susceptibility to post-COVID syndrome. There is also early evidence that cognitive training could improve the cognitive symptoms of post-COVID syndrome in domains like attention, memory, reasoning, perception and coordination [132]. In the current study, our focus was on older females (aged 60–75 years) with post-COVID syndrome because they are more likely to develop chronic symptoms than males, have a different disease course and are understudied in this context. We first compared female patients with post-COVID syndrome to similarly aged male patients, to highlight sex differences in brain dynamics before an adaptive task-switching training protocol. Additionally, we investigated the course of training-related changes in brain dynamics in both groups to detect if cognitive training is similarly effective in both females and males. We then compared our female patients to similarly aged neurotypical females, to examine post-COVID-related alterations in brain function before cognitive training and training effects. We hypothesized that the age-related shift from increasingly global to increasingly local neural processing would be more pronounced in the post-COVID group, but this difference could be attenuated by cognitive training.

## Materials and methods

### Participants

The demographic data of our experimental groups can be seen in Table 1. Our data was collected in two phases. The healthy old-training group (20 participants, all females) was recruited before the COVID-19 pandemic; thus, they did not encounter the virus. The post-COVID old-training group (30 participants, 14 males, 16 females) was registered between 2021 and 2022. The inclusion criteria for the post-COVID group were COVID-19 infection within 6 months prior to participation (established through quick test or PCR test), successful remission from the acute phase of the infection and persistence of physical, cognitive and/or neurological symptoms at least for 8 weeks after remission which were still present at the beginning of training. People who were hospitalized during their acute phase were excluded. All of our participants had mild to moderate flu-like symptoms during the acute phase of the infection which lasted from a few days to 2 weeks in average. In our study, the main focus was on the cognitive and neurological post-COVID symptoms like fatigue, memory, concentration and attentional problems, sleep problems, dizziness, anosmia and ageusia. All of our post-COVID participants reported some of these symptoms in a mild to moderate form where these were not having seriously debilitating effect in our participants’ everyday life, but they detected subjective decrease in life quality and mental performance. All participants had normal, or corrected-to-normal vision, did not have any neurological or psychiatric disorders (before the COVID-19 infection in the post-COVID group) and did not take any antidepressant or neuropsychiatric medication.

**Table 1:** Descriptive statistics (mean ± SD) of age, IQ-score and behavioural measures of the trained task-switching for our three experimental groups in pre- and post-training. *RT_TS* reaction time in ms for switch trials, *RT_TR* reaction time in ms for repeat trials, *HR_TS* hit rate for switch trials, *HR_TR* hit rate for repeat trials, MC mixing costs in ms; *SC* switching costs in ms

	Healthy old female (HF, *N* = 20)		Post-COVID old female (CF, *N* = 16)		Post-COVID old male (CM, *N* = 14)	
	Pre (1)	Post (2)	Pre	Post	Pre	Post
AGE	65.3 ± 3.3	-	65.8 ± 3.2	-	66.8 ± 4.8	-
IQ	117.9 ± 16.7	-	131.3 ± 19.8	-	136.8 ± 23.1	-
RT_TS	1076.77 ± 154.20	728.23 ± 124.76	1129.44 ± 154.48	863.71 ± 111.70	1155.24 ± 135.57	932.54 ± 132.48
RT_TR	1057.97 ± 142.87	711.18 ± 121.78	1108.30 ± 139.44	839.45 ± 115.05	1141.16 ± 155.11	910.74 ± 132.11
HR_TS	0.665 ± 0.161	0.934 ± 0.041	0.682 ± 0.139	0.904 ± 0.084	0.706 ± 0.172	0.910 ± 0.078
HR_TR	0.673 ± 0.147	0.944 ± 0.030	0.709 ± 0.151	0.948 ± 0.058	0.706 ± 0.204	0.933 ± 0.072
MC	316.59 ± 114.20	116.55 ± 70.23	277.66 ± 114.80	107.24 ± 62.74	279.98 ± 164.29	165.03 ± 84.05
SC	18.80 ± 64.74	17.05 ± 42.42	21.15 ± 56.61	24.26 ± 62.02	14.08 ± 74.74	21.80 ± 54.40

We recruited our participants for the healthy old-training group through our database and for the post-COVID old-training group through online news portals and Facebook. Written informed consent was obtained from all participants, and they were paid for their contribution. The study was approved by the National Institute of Environmental Health, Hungary, and was carried out in accordance with the Declaration of Helsinki.

### Procedure

At the start of the experiment, post-COVID participants underwent a neurological examination to exclude potential organic causes for self-reported post-COVID symptoms and to assess general neurological functioning (Bajcsy-Zsilinszky Hospital, Budapest, Hungary). All participants then completed a cognitive training protocol. EEG and behavioural data were recorded before and after 1 month (eight sessions) of adaptive cognitive training on an informatively cued task-switching paradigm. A detailed description and technical information about the cognitive training protocol and the applied cognitive tasks can be found in our previous study [121]. In this paper, we focus on the training effect on intrinsic neural dynamics and provide only a brief description of the cognitive training, behavioural results of the trained task and the resting-state EEG data.

During the pre-training session in the laboratory, each participant’s health status, habits, demographic data and IQ were documented. We measured participants’ IQ by the Hungarian version of the Wechsler Adult Intelligence Scale (WAIS-IV, [133, 134]) to detect potential post-COVID-related cognitive changes (IQ scores can be found in Table 1). Post-COVID symptoms and related information (e.g. the approximate date of COVID-19 infection, the length of the acute phase, vaccination) were registered in the post-COVID group through self-report questionnaires.

All participants performed several different tasks during the pre- and post-training session during EEG recording, in the following order: (1) eyes open resting-state for 2 min each (the focus of this study), (2) informatively cued task-switching paradigm with no-go stimuli—letter classification and parity task (trained task), (3) informatively cued task-switching paradigm—colour and shape classification (near-transfer task measuring similar cognitive domain as in the trained task but with different stimuli) and (4) attentional network test (far-transfer task measuring different cognitive domain as in the trained task, in this case, attentional processes). Each task began with a practice block where the participants were given the instructions both orally and in writing. Between the two laboratory sessions, all participants completed eight 1-h-long adaptive training sessions of the trained task during a 1-month-long period. During every session of the adaptive training, the difficulty level of the task was personalized in each block based on the current performance of each participant.

In the current study, we analysed the pre-training and post-training eyes open resting-state EEG data for detecting intrinsic neural dynamics and behavioural performance on the trained task as a measure for the effectiveness of cognitive training in healthy old females, post-COVID old females and post-COVID old males.

### Measurement and analysis of behavioural data from the trained task

In order to interpret the behavioural data from the trained task sufficiently, we briefly introduce this task procedure. Every trial started with a coloured cue (fixation cross) where yellow and orange cues were assigned to the letter classification task (vowel/consonant decision) and blue and green cues to the number classification task (odd/even decision). After 700 ms, a letter-number target pair was shown in the centre of the screen. Participants responded by pressing a button of a game pad with their left or right index finger based on the mapping of the letter and parity task which was counterbalanced between participants. In all trials except four per block, the two stimuli required opposite button presses. In 25% of the trials, targets were special characters in which case response had to be withheld (no-go trial). The target was shown until response (maximum 2000 ms) followed by a feedback which was displayed on the screen for 200 ms. The paradigm had the following restrictions: (1) cue colour was never repeated in successive trials, (2) target stimuli were presented in pseudorandom order with no repetition on successive trials, (3) 50% switch probability, (4) two no-go trials could not follow each other, (5) maximum three switch (different task as in previous trial) or repeat (same task as in previous trial) trials could follow each other. Two blocks of single letter and two blocks of single number classification trials were presented at the beginning of the task, which were followed by 10 blocks of mixed trials (50 trials per block). In this study, we excluded the no-go effect and focused on the go-after-go trials.

For the behavioural measures, we used reaction times (RTs) and hit rates (HRs; ratio of correct responses) for repeat and switch trials, as well as two specific indices related to the task-switching paradigm: mixing costs (MCs) and switching costs (SCs). MC is the reaction time difference between repeat trials in mixed-task blocks (two task-sets) and the trials in single-task blocks (one task-set). It manifests in slower responses in mixed-task trials, which reflects extra working memory demand in this condition. SC is the reaction time difference between switch and repeat trials, which generally shows slower responses in switch trials and is connected to cognitive flexibility [135, 136].

For the statistical analyses, we applied repeated measures of ANOVA for RT, HR, MC and SC data separately with *Group* (post-COVID old females/post-COVID old males and healthy old females/post-COVID old females comparisons separately) as a between-subject factor and *Training* (pre-training/post-training) as a within-subject factor. Partial eta squared (*η*^2^_*p*_) was used for calculating effect size. We focused on *Training* main effects, *Training* × *Group* interactions and their post hoc analyses which were performed using Tukey’s honestly significant difference (HSD). All analyses were performed in Statistica 13 (TIBCO Software Inc., Palo Alto, CA, USA).

### EEG recording and pre-processing

Electrophysiological recording was performed in an electrically and acoustically shielded room. Continuous EEG was recorded by EasyCap active electrode system with ActiChamp amplifier (band pass: DC-70 Hz) using BrainVision Recorder 1.21.0303 software (BrainProducts GmbH, Gilching, Germany; sampling rate: 1000 Hz). Thirty-five passive Ag/AgCl electrodes were placed on Fp1, AFz, Fp2, F7, F3, Fz, F4, F8, FT9, FC5, FC1, FC2, FC6, FT10, T7, C3, Cz, C4, T8, TP9, CP5, CP1, CP2, CP6, TP10, P7, P3, Pz, P4, P8, PO9, O1, Oz, O2 and PO10, referenced to the tip of the nose, with FCz as ground. Vertical and horizontal eye movements were recorded by electrodes placed above and below the left eye (VEOG) and in the outer canthi of the eyes (HEOG). The impedance of the electrodes was kept below 10 kΩ.

For pre-processing raw resting-state EEG data offline for further analyses, functions of EEGLAB v.14.1.0b [137] were applied for finite impulse response band-pass filtering at 0.5–40 Hz followed by independent component analysis (ICA) for removing ocular (i.e. eye blinks, horizontal eye movements), muscle and cardiac artefact. Data were segmented into 2500-ms epochs continuously, and the epochs were rejected if they had a voltage change larger than 100 µV between their minimum and maximum.

### Measuring neural dynamics from pre-processed resting-state EEG data

As our metric of resting-state neural dynamics in pre- and post-training conditions, we applied multiscale entropy (MSE) for measuring intrinsic signal complexity and spectral power density (SPD) for extracting intrinsic oscillatory activity as complementary analysis. Detailed descriptions of these methods and the procedure for selecting specific electrodes for these analyses can be found in our previous study [131]; thus, we will give only a brief summary in the current paper.

MSE measures EEG signal complexity by calculating entropy at different timescales, where greater MSE values represent greater entropy and thus greater unpredictability [85, 86]. This method calculates sample entropy for scale-length averaged time series in each epoch by detecting repetitive patterns based on pattern length (*m*, how many data points are used for pattern matching, we used m = 2 based on [138]) and tolerance level (*r*, maximal absolute amplitude difference between two data points for considering them matching, we used *r* = 0.5 based on [139]). Therefore, in our case sample, entropy reflects the probability that two sequences that match on the first two data points will also match on the next data point. We calculated MSE for each epoch, and each participant for timescales from 1 to 50 ms using the algorithm available at www.physionet.org/physiotools/mse/ and calculated average MSE values for each timescale and electrode in every participant separately in the pre- and post-training conditions.

Spectral power density (SPD) extracts the frequency content of the EEG signal (in our case with 1000 Hz/2500 time point = 0.4 Hz resolution) applying fast Fourier transform on single-trial data and measures the relative contributions of different frequency bands to the total spectral power on normalized data. Single-trial estimates were averaged across all trials for each frequency and each electrode in every participant separately to obtain mean SPD for pre- and post-training conditions. These two metrics of neural dynamics complement each other in order to detect if the sex-, post-COVID- and training-related differences in the EEG data are driven more by linear (SPD) or nonlinear (MSE) dependencies [104].

Statistical analyses were performed with partial least squares (PLS) [140–143]. It is a data-driven multivariate technique which operates on the entire data structure at once, extracting the patterns of maximal covariance between brain signals (MSE/SPD), groups (healthy old female/post-COVID old male/post-COVID old female) and conditions (pre-training/post-training) through a new set of variables, namely latent variables (LVs). Each LV contains three vectors: design saliences (the degree to which each condition within each group is related to the identified brain signal pattern), electrode saliences (the weight of electrodes and timescales/frequencies that are most related to the group and condition effects in the identified signal pattern) and singular values (the strength of covariance between the group and condition contrasts and signal pattern). Statistical assessment in PLS is performed across two levels: permutation testing is applied for assessing the overall significance level of each LV [144], and bootstrap resampling is used to assess the relative contribution and stability of the electrode weights in the detected signal pattern [145, 146]. Five hundred permutations and 500 bootstrap samples were tested for every analysis, and the Scientific colour map vik [147] was used for data visualization.

## Results

### Behavioural results

The neurological examination did not reveal any organic nervous system abnormalities corresponding to the complaints reported by the post-COVID participants. These complaints included neurocognitive symptoms (such as fatigue, memory issues, concentration and attention problems, sleep disturbances, dizziness, headache, anosmia or ageusia), cardiorespiratory symptoms (such as coughing, high pulse rate) and other symptoms (e.g. muscle and joint pain, digestive problems), none of which showed significant improvement during cognitive training.

To examine the direct behavioural influence of cognitive training, we compared the IQ-scores and the performance in the applied and trained task-switching paradigm between post-COVID old females (CF) and old males (CM) for detecting sex-related differences and between healthy old females (HF) and post-COVID old females (CF) for detecting the effect of post-COVID syndrome. Details about the descriptive statistics of the behavioural variables can be seen in Table 1.

Regarding the effect of sex in post-COVID syndrome, our CF and CM groups did not differ in age (*t*(28) =  − 0.666, *p* = 0.511, Cohen’s *d* = 0.24) or IQ-score (*t*(28) =  − 0.707, *p* = 0.486, Cohen’s *d* = 0.26). Comparing the behavioural indices of the trained task, both sex-groups showed training-related improvement in RT (faster responses; *Training* main effect: *F*(1,28) = 105.634, *p* < 0.001, *η*^2^_*p*_ = 0.79), HR (more correct responses; *training* main effect: *F*(1,28) = 79.592, *p* < 0.001, *η*^2^_*p*_ = 0.74) and MC (lower mixing costs; *training* main effect: *F*(1,28) = 33.660, *p* < 0.001, *η*^2^_*p*_ = 0.55), but not in SC (*Training* main effect: *F*(1,28) = 0.177, *p* = 0.677, *η*^2^_*p*_ = 0.01). However, the two sex-groups did not differ in their performance before or after training (all *training* × *group* interactions and corresponding post hoc tests were not significant).

Regarding the effect of post-COVID, our HF and CF groups did not differ in age (*t*(34) =  − 0.465, *p* = 0.645, Cohen’s *d* = 0.16); however, the CF group had significantly higher IQ than the HF group (*t*(34) =  − 2.200, *p* = 0.035, Cohen’s *d* = 0.73). Comparing the behavioural indices of the trained task, both groups showed training-related improvement in RT (faster responses; *training* main effect: *F*(1,34) = 288.528, *p* < 0.001, *η*^2^_*p*_ = 0.89), HR (more correct responses; *training* main effect: *F*(1,34) = 139.025, *p* < 0.001, *η*^2^_*p*_ = 0.80), and MC (lower mixing costs; *training* main effect: *F*(1,34) = 88.868, *p* < 0.001, *η*^2^_*p*_ = 0.72), but not in SC (*training* main effect: *F*(1,34) = 0.003, *p* = 0.954, *η*^2^_*p*_ < 0.01). While both groups showed similar performance before training (all indices’ *training* × *group* interactions and corresponding post hoc ps were not significant), the HF group was significantly faster compared to CF group after the training (*training* × *group* interaction for RT: *F*(1,34) = 4.929, *p* = 0.033, *η*^2^_*p*_ = 0.13; (HF_after − CF_after) post hoc *p* = 0.022), but they did not differ in other indices (*training* × *group* interactions for HR, MC, SC and corresponding post hoc ps were not significant).

### Sex differences in the effect of post-COVID syndrome and cognitive training

We were interested if post-COVID syndrome had different effects on the intrinsic neural dynamics of older female versus male participants and if the cognitive training is comparably suitable and effective in both sex groups for altering resting-state neural processing in post-COVID syndrome. Therefore, task PLS analyses were applied with group (post-COVID female/male) and training condition (pre/post) levels on MSE and SPD data.

**Fig. 1:**
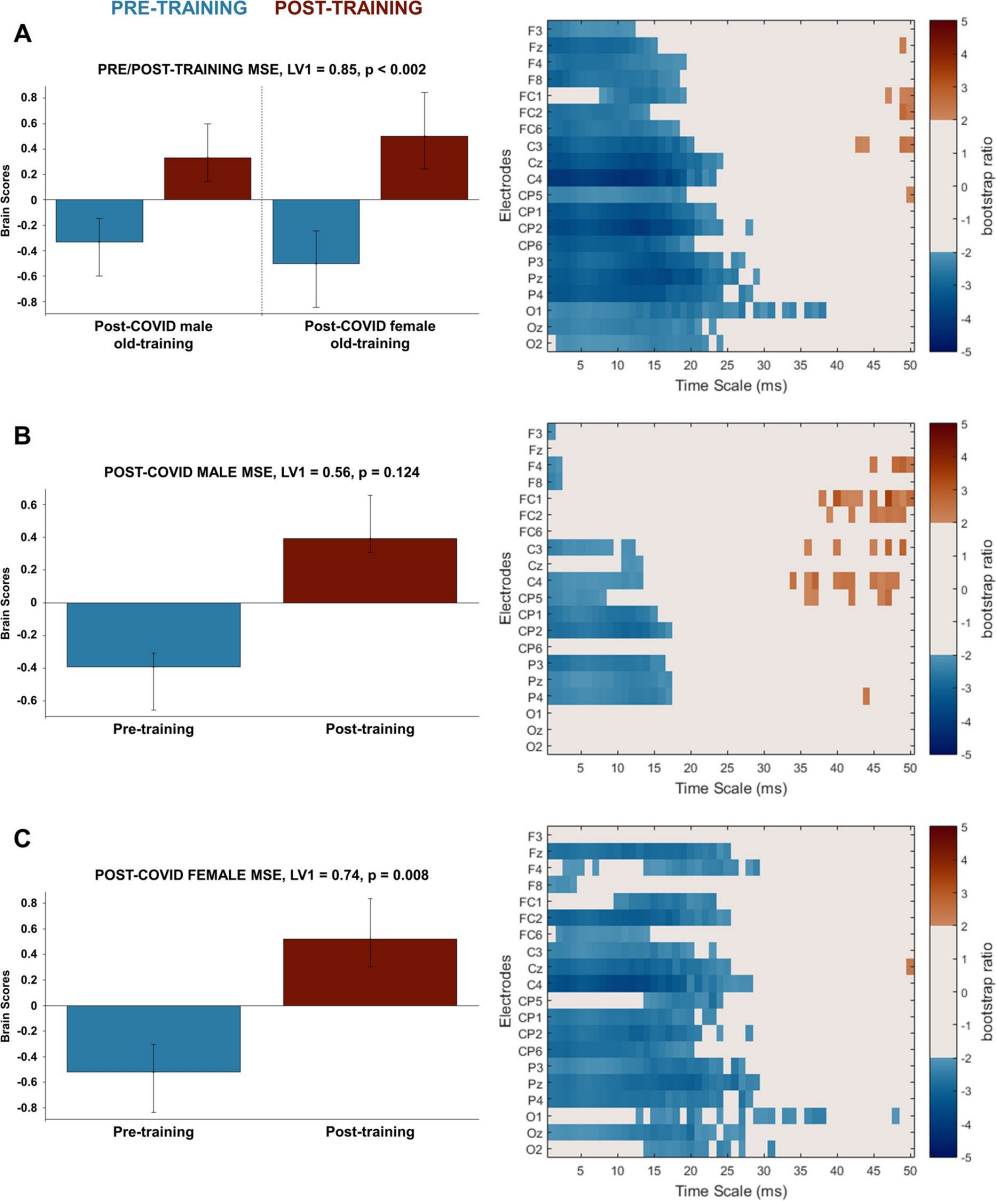
PLS analysis results for the effect of gender and cognitive training in post-COVID syndrome on resting-state MSE in older adults. **A** The significant contrast and interaction between experimental groups (old male/female post-COVID) and training conditions (pre-training/post-training); **B** the not significant contrast between pre- and post-training conditions in post-COVID old male group; and **C** the significant contrast between pre- and post-training conditions in post-COVID old female group. On the left side of every panel, the bar graph depicts the contrast that was significantly expressed across electrodes and timescales as determined by permutation tests, with error bars denoting 95% confidence intervals. On the right side of every panel, the bootstrap ratio map illustrates the electrodes and timescales at which the contrast displayed in the bar graphs was significant. Values represent the ratio of the individual electrode weights and the bootstrap-derived standard error (thresholded at 2.0 which corresponds approximately to *p* < 0.05) where positive values are plotted in warm colours and negative values in cool colours. In **A**, positive values indicate timescales and electrodes showing increases from pre- to post-training condition while negative values indicate timescales and electrodes showing decreases from pre- to post-training condition in both the old male and old female post-COVID groups in resting-state MSE. In **B**, positive values indicate timescales and electrodes showing not significantly increased resting state MSE while negative values reveal not significantly decreased resting state MSE from pre- to post-training condition in old male post-COVID group. In **C**, positive values indicate timescales and electrodes showing increased resting-state MSE while negative values reveal decreased resting-state MSE from pre- to post-training condition in the old female post-COVID group

Since it has been suggested by previous literature that post-COVID syndrome could differently affect sex groups, we first compared the intrinsic brain dynamics in post-COVID old females and males to determine if there are differences between them prior to cognitive training. For multiscale entropy, we found no significant differences in intrinsic neural complexity before training in our similarly aged sex groups (LV1, singular value = 0.55, *p* = 0.561). In order to identify if cognitive training modifies intrinsic neural dynamics in a similar way in female and male post-COVID patients, we ran a PLS analysis with both sex-groups and training conditions on MSE data. Additionally, to detect the specific effects of cognitive training in our post-COVID groups, we ran the PLS analysis on pre- and post-training data on post-COVID females and males separately. The PLS analysis examining pre- and post-training in both sexes identified one significant latent variable showing a similar pattern of pre- to post-training changes in females and males with post-COVID syndrome (LV1, singular value = 0.85, *p* < 0.002, Fig. 1A): fine and middle scale entropy (1–25 ms) decreased from pre- to post-training condition in both sex groups. However, this pattern was being driven by females to a higher extent, as confirmed by the larger design scores in post-COVID females (± 0.59) compared to males (± 0.39) (Fig. 1A). The results from our separate sex-group PLS analyses supported this finding: the training-related change in resting-state neural complexity was significant only in the old female group, as shown in the single significant latent variable in the separate task PLS analysis for this post-COVID group (for females, LV1 singular value = 0.74, *p* = 0.008; for males, LV1 singular value = 0.56, *p* = 0.124, Fig. 1B,C). For females, the training-related decrease in MSE was observed in finer timescale entropy (1–25 ms) while coarse timescale entropy (30–50 ms) remained unchanged.

**Fig. 2:**
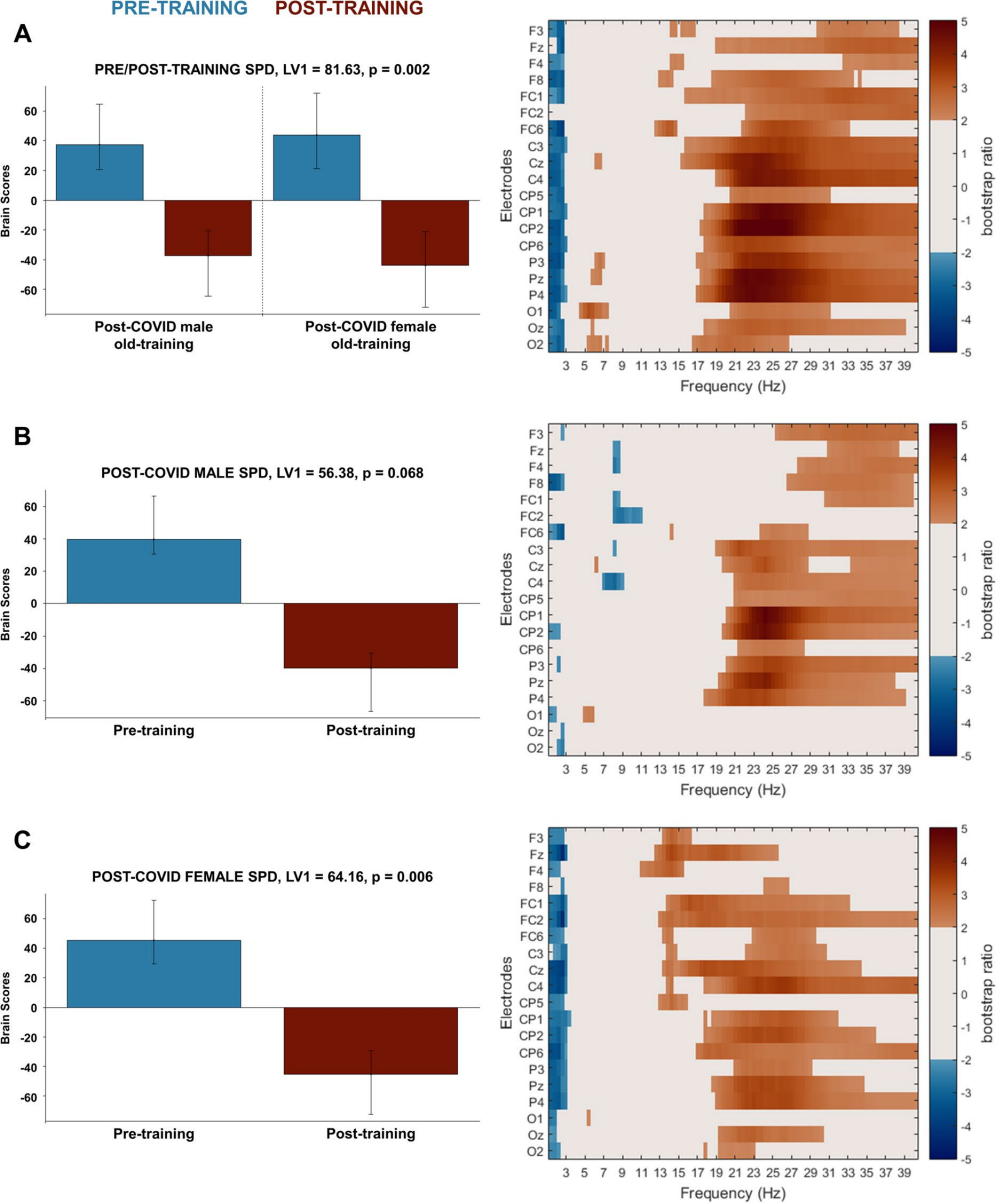
PLS analysis results for the effect of gender and cognitive training in post-COVID syndrome on resting-state SPD in older adults. **A** The significant contrast and interaction between experimental groups (old male/female post-COVID) and training conditions (pre-training/post-training); **B** the not significant contrast between pre- and post-training conditions in post-COVID old male group; and **C** the significant contrast between pre- and post-training conditions in the post-COVID old female group. On the left side of every panel, the bar graph depicts the contrast that was significantly expressed across electrodes and frequencies as determined by permutation tests, with error bars denoting 95% confidence intervals. On the right side of every panel, the bootstrap ratio map illustrates the electrodes and frequencies at which the contrast displayed in the bar graphs was significant. Values represent the ratio of the individual electrode weights and the bootstrap-derived standard error (thresholded at 2.0 which corresponds approximately to *p* < 0.05) where positive values are plotted in warm colours and negative values in cool colours. In **A**, positive values indicate frequencies and electrodes showing decreases from pre- to post-training while negative values indicate frequencies and electrodes showing increases from pre- to post-training in both the old female and old male post-COVID groups in resting-state SPD. In **B**, positive values indicate frequencies and electrodes showing not significantly decreased resting-state MSE while negative values reveal not significantly increased resting-state SPD from pre- to post-training condition in the old male post-COVID group. In **C**, positive values indicate frequencies and electrodes showing decreased resting-state SPD while negative values reveal increased resting-state SPD from pre- to post-training condition in the old female post-COVID group

To bolster and complement our complexity results, we ran PLS analyses on spectral power density data in the same manner as on multiscale entropy data, and we found similar changes by sex and cognitive training. We did not detect significant difference in intrinsic neural oscillatory activity before cognitive training in our similarly aged post-COVID sex-groups (LV1, singular value = 55.77, *p* = 0.535). The PLS analysis examining pre- and post-training in both sexes identified one significant LV for SPD data showing a similar direction of training-related change in our female and male post-COVID participants (LV1, singular value = 81.63, *p* = 0.002, Fig. 2A): higher frequency band power (15–40 Hz) decreased while very low frequency band power (1–3 Hz) increased with cognitive training. Again, this pattern was being driven by females to a higher extent as indicated by the larger design scores in post-COVID females (± 0.54) compared to males (± 0.46). The results from our separate sex-group PLS analyses supported this finding: the training-related change in resting-state neural oscillatory activity was significant only in the old female group, as shown in the single significant latent variable in the separate task PLS analysis for this post-COVID group (for females, LV1 singular value = 64.16, *p* = 0.006; for males, LV1 singular value = 56.38, *p* = 0.068, Fig. 2B,C). More precisely, old female post-COVID participants showed training-related decrease in high alpha band power (11–14 Hz) at fronto-central areas, in widespread beta band power (14–30 Hz) and in more sparsely distributed low gamma power (30–40 Hz) as well as general increase in delta band power (1–3 Hz).

### Differences between females with post-COVID-syndrome and controls pre- and post-cognitive training

In addition to sex differences, we examined if older adults with post-COVID syndrome show different intrinsic brain dynamics compared to their healthy peers prior to cognitive training and if these differences can be diminished with this intervention method. Since we found sex-related differences in the training effect and we only had healthy training controls for older females, we selectively compared the female post-COVID training group with similarly aged controls who went through the same cognitive training process. Thus, task PLS analysis was applied to examine group (healthy/post-COVID old female) differences in pre- and post-training conditions separately for MSE and SPD data.

**Fig. 3:**
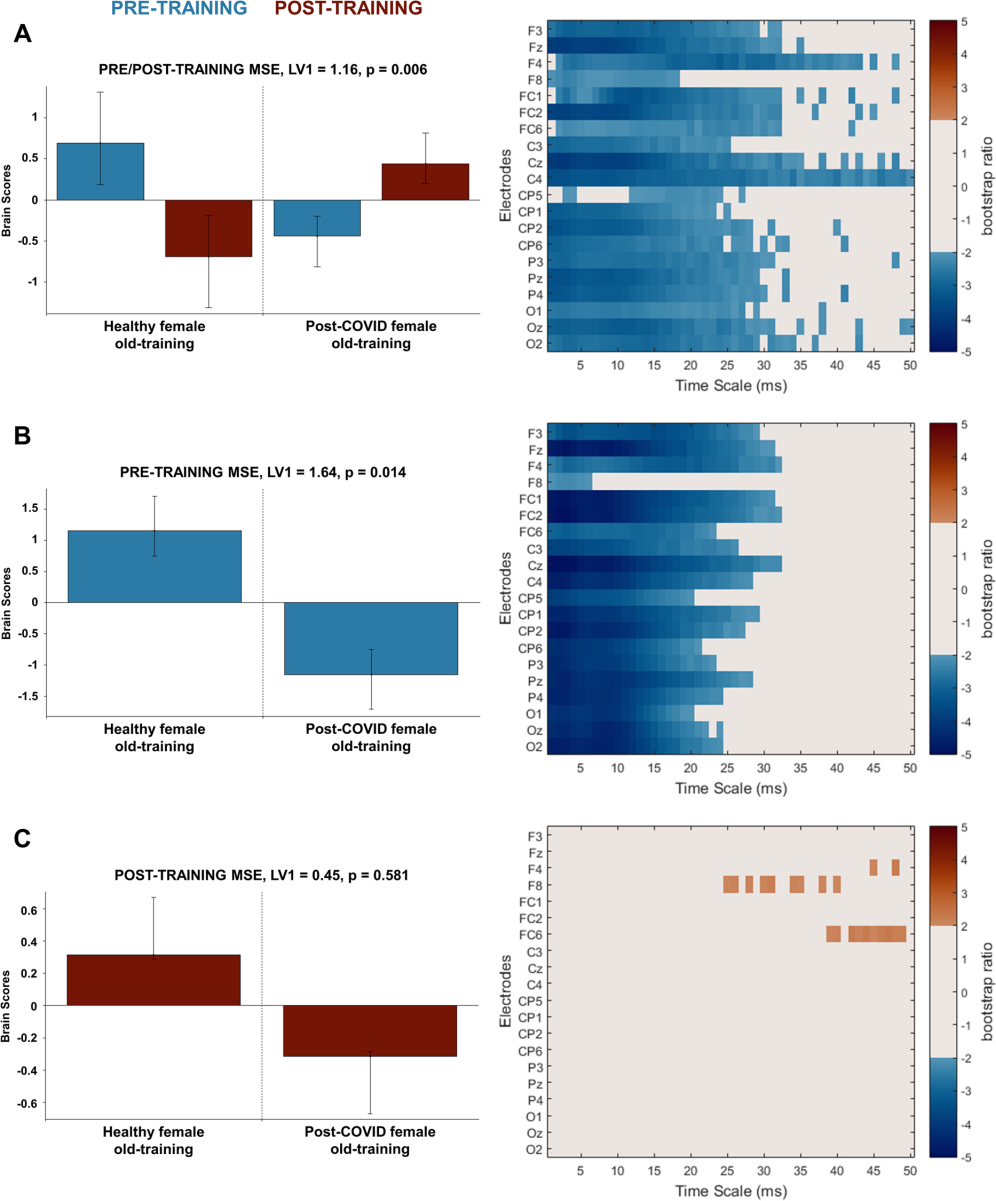
PLS analysis results for the effect of post-COVID syndrome and cognitive training on resting-state MSE in older female adults. **A** The significant contrast and interaction between experimental groups (healthy/post-COVID old female) and training conditions (pre-training/post-training); **B** the significant contrast between healthy and post-COVID old female groups before cognitive training; and **C** the lack of significant contrast between healthy and post-COVID old female groups after cognitive training. On the left side of every panel, the bar graph depicts the contrast that was significantly expressed across electrodes and timescales as determined by permutation tests, with error bars denoting 95% confidence intervals. On the right side of every panel, the bootstrap ratio map illustrates the electrodes and timescales at which the contrast displayed in the bar graphs was significant. Values represent the ratio of the individual electrode weights and the bootstrap-derived standard error (thresholded at 2.0 which corresponds approximately to *p* < 0.05) where positive values are plotted in warm colours and negative values in cool colours. In **A**, negative values indicate timescales and electrodes showing increases in healthy old female group and decreases in post-COVID old female group from pre- to post-training in resting state MSE. In **B**, negative values indicate timescales and electrodes showing increased resting-state MSE in the post-COVID old female group compared to the healthy old female group in the pre-training condition. In **C**, the contrast between healthy and post-COVID old female groups in resting state MSE is not significant post-training

Regarding multiscale entropy, we found different patterns of change in our healthy and post-COVID female groups through the significant latent variable (LV1, singular value = 1.16, *p* = 0.006, Fig. 3A). Looking at the pre-training MSE data, we identified widespread increases in fine and middle scale entropy (1–30 ms) in the post-COVID old female group compared to the healthy one which was revealed by the one significant latent variable (LV1, singular value = 1.64, *p* = 0.014, Fig. 3B). However, these differences disappeared with cognitive training as revealed by the post-training MSE data where there was no significant latent variable; thus, no significant difference was found between the healthy and post-COVID old female groups (Fig. 3C).

**Fig. 4:**
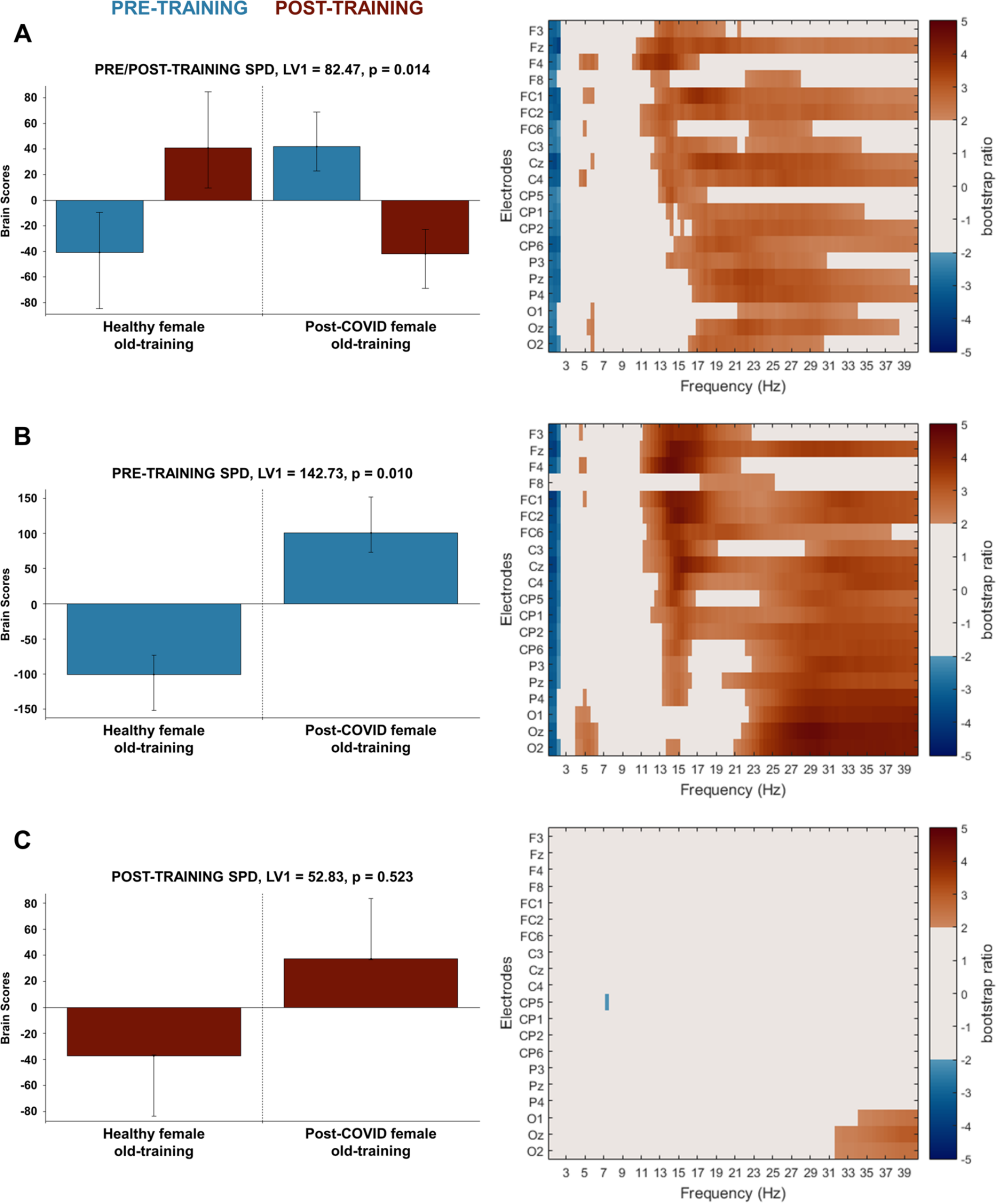
PLS analysis results for the effect of post-COVID syndrome and cognitive training on resting-state SPD in older female adults. **A** The significant contrast and interaction between experimental groups (healthy/post-COVID old female) and training conditions (pre-training/post-training); **B** the significant contrast between healthy and post-COVID old female groups before cognitive training; and **C** the lack of significant contrast between healthy and post-COVID old female groups after cognitive training. On the left side of every panel, the bar graph depicts the contrast that was significantly expressed across electrodes and frequencies as determined by permutation tests, with error bars denoting 95% confidence intervals. On the right side of every panel, the bootstrap ratio map illustrates the electrodes and frequencies at which the contrast displayed in the bar graphs was significant. Values represent the ratio of the individual electrode weights and the bootstrap-derived standard error (thresholded at 2.0 which corresponds approximately to *p* < 0.05) where positive values are plotted in warm colours and negative values in cool colours. In **A**, positive values indicate frequencies and electrodes showing increases in healthy old female group and decreases in post-COVID old female group from pre- to post-training in resting-state SPD, while negative values indicate frequencies and electrodes showing decreases in healthy old female group and increases in post-COVID old female group from pre- to post-training in resting state SPD. In **B**, positive values indicate frequencies and electrodes showing increased resting-state SPD in post-COVID old female group compared to healthy old female group in pre-training condition, while negative values indicate frequencies and electrodes showing decreased resting-state SPD in the post-COVID old female group compared to the healthy old female group in pre-training condition. In **C**, the contrast between the healthy and post-COVID old female groups in resting state SPD is not significant post-training

Analysing the spectral power density data in the same manner, we found similar changes by cognitive training as in the MSE data which complement our complexity findings. Different pattern changes were found in our healthy and post-COVID old female groups through the significant latent variable (LV1, singular value = 82.47, *p* = 0.014, Fig. 4A). Examining the pre-training SPD data, we identified widespread increase in higher frequency band power (11–40 Hz) and widespread decrease in very low–frequency band power (1–2 Hz delta power) in the post-COVID old female group compared to the healthy one which was revealed by the one significant latent variable (LV1, singular value = 142.73, *p* = 0.010, Fig. 4B). More precisely, the upper alpha band (11–14 Hz) was increased more frontally, while upper beta (21–30 Hz) and lower gamma (30–40 Hz) bands were increased at more posterior areas in the post-COVID group. However, these differences disappeared with cognitive training as revealed by the post-training SPD data where there was no significant latent variable; thus, no significant difference was found between the healthy and post-COVID old female groups (Fig. 4C).

**Fig. 5:**
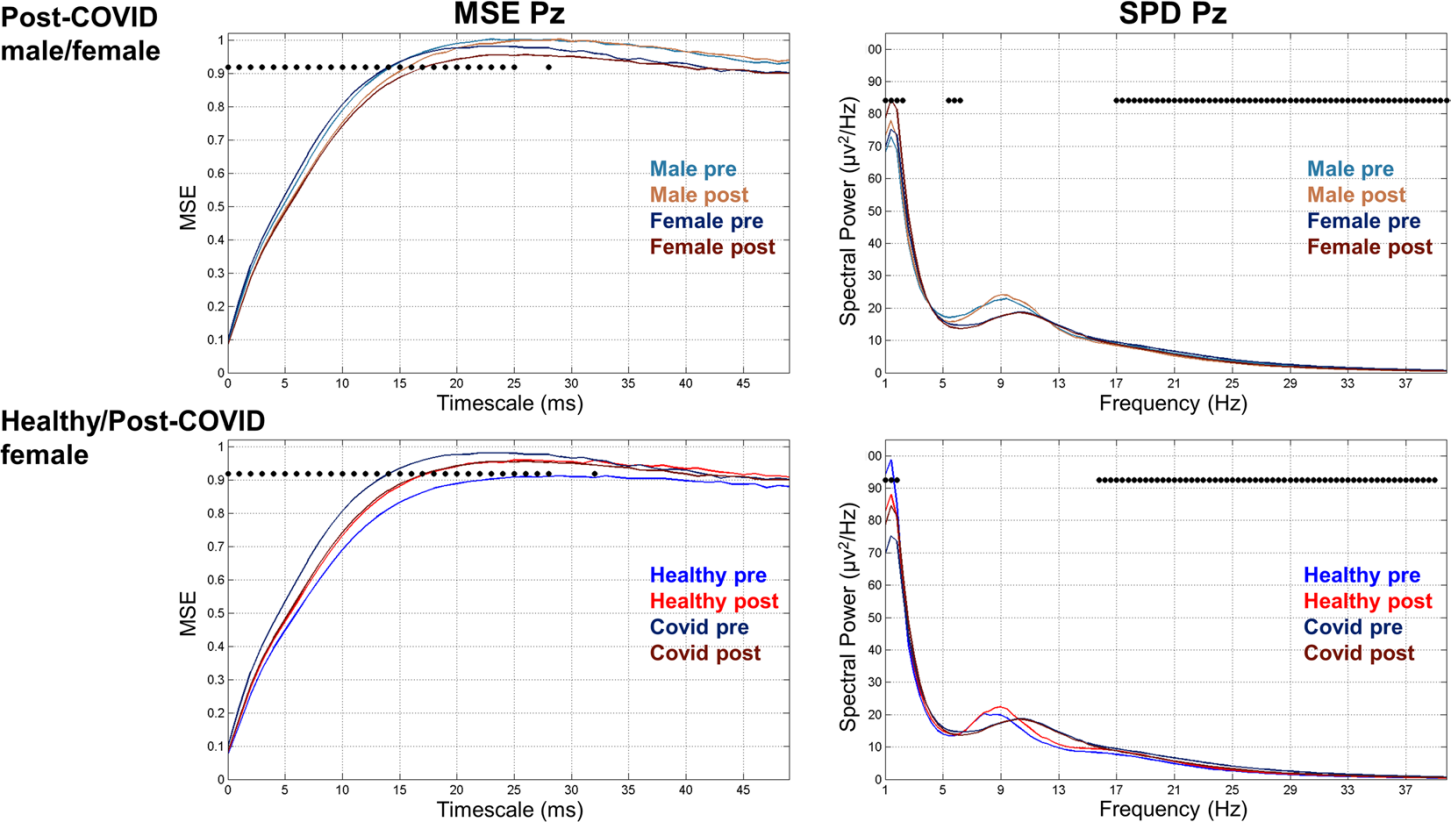
The timescale and frequency changes at Pz electrode in MSE (left) and SPD (right) data, respectively, in our post-COVID old male/female (top) and healthy/post-COVID old female (down) comparisons between pre- and post-training conditions. The black dots represent those timescales and frequencies where the 2 × 2 (groups × conditions) PLS analyses showed significant effect in the significant latent variable (LV1)

To ease interpretation of our results, a representative electrode illustrating the course of MSE and SPD changes for both of our comparisons (post-COVID male/female, healthy/post-COVID female) is presented at electrode Pz in Fig. 5.

In our main analyses, we focused separately on how the resting-state neural dynamics differ between female/male and healthy/patient participants in post-COVID syndrome in older age group. For the sake of comprehensiveness, we provide three group analyses (healthy old female/post-COVID old female/post-COVID old male) on pre- and post-training MSE and SPD data in the Supplementary Materials. These results are consistent with findings and conclusions of our main analyses.

## Discussion

In this exploratory study, we investigated how post-COVID syndrome influences intrinsic brain dynamics. Given previous studies indicating a higher sensitivity to post-COVID syndrome in women compared to men [30–36], we analysed the intrinsic brain dynamics in post-COVID old females and males to determine if there are differences between them, thus warranting separate approaches in their treatment. Furthermore, we compared how resting-state neural processing differed between older women with post-COVID syndrome and healthy female controls and if cognitive training could influence these differences.

Although the prevalence of post-COVID syndrome may vary between sexes, we did not find any sex-related differences in brain dynamics before training, suggesting that the virus affected both females and males similarly (at least in an older adult sample with similarly high IQ-score and symptomology). We did, however, observe differences following the adaptive task-switching training. While both sexes gained similar training-induced improvements in task performance (faster RT, increased correct responses and lower mixing costs related to better working memory function), only females showed significant training-related changes in intrinsic information processing, whereas males did not. More precisely, in post-COVID females, adaptive task-switching training was associated with a widespread attenuation in the shift to local information processing as seen through decreased fine and middle scale entropy as well as higher frequency band power from pre- to post-training. Even though our MSE timescales did not extend to frequencies within the delta band, we did find an additional widespread power increase within this range from pre- to post-training. These results imply that the chronic phase of COVID-19 infection had similar effects on intrinsic neural communication in both sexes, but cognitive training modulated post-COVID-related neural changes specifically in females.

There is only sparse previous literature about general sex differences in the effectiveness of cognitive training and its functional mechanisms in the brain. Some studies have revealed greater improvements for females as compared to males on attentional processes by executive function or temporary memory training [148] and in cognitive training for patients with mild cognitive impairment (MCI) [149]. More work has examined sex differences in age-related neurodegenerative conditions like MCI and Alzheimer’s disease [150–153], showing that females are more likely to develop more extensive brain damage and more severe symptoms. Males, on the other hand, maintain stronger and more intact functional connectivity between the left and right hippocampus, precuneus, brain stem and other regions, and this appears to have a neuroprotective effect [154, 155]. Overall, these studies suggest that females are more susceptible to age-related neurodegenerative diseases, showing greater disruptions in brain structure and function as compared to males. Contrary to this previous work, we did not find sex-related differences in intrinsic neural dynamics for this condition. However, neural dynamics measures during post-COVID-syndrome were more effectively modulated by cognitive training in females. Consequently, these results emphasize the importance of sex-specific medical treatments and neuropsychological interventions in the future regarding post-COVID syndrome, neurodegenerative disorders and general age-related changes.

We also compared resting-state complexity (MSE) and oscillatory (SPD) measures between healthy and post-COVID old females prior to cognitive training. We found greater complexity associated with local information processing in female patients compared to their healthy peers, while their task-switching performance did not differ significantly. This pattern is similar to the age-associated shift from global to local processing in healthy aging [83, 91, 93, 131] and may be connected to the hypothesized accelerated neural aging processes in post-COVID syndrome [41]. However, the decreased emphasis and flexibility of distributed neural processing was less evident in our post-COVID old female group since we only detected decreased low-frequency band power but not decreased coarse scale entropy. This line of evidence suggests that the efficiency and adaptability of neural communication showed less change in large-scale brain dynamics and more complex changes for local neural processing with post-COVID in our older female participants.

All of our experimental groups, but especially the post-COVID ones, had very high intelligence based on WAIS-IV IQ-scores. High intelligence is a key factor in maintaining a high cognitive reserve against aging and disorder-related cognitive decline [156–161]. Therefore, it is plausible that the increased complexity of local neural dynamics and information processing capacity in post-COVID old females is a compensatory mechanism [162–164]. This is suggested by their similar behavioural performance in the cognitively demanding task-switching task compared to their healthy peers. Previous studies have shown that people with disrupted cognitive functioning but high cognitive reserve can achieve similar performance to their healthy peers. However, they require more cognitive effort to achieve the same level of performance and cannot maintain this high level of performance for extended periods [165]. Consequently, this heightened neural effort may be represented by the increased capacity of local information processing in order to achieve similar behavioural performance in the post-COVID female group. As for why this hypothesized compensatory mechanism is detected in local intrinsic neural processing capacity and not in large-scale processing flexibility, one possibility is the less efficient large-scale neural dynamics in post-COVID syndrome. This inefficiency may be connected to chronic COVID-19-induced accelerated aging through neuroinflammation, which causes decreased synaptic connections and plasticity due to microglia reactivity, degraded axon myelination due to astrocyte reactivity [16–18] and chronic (cerebro)vascular and endothelial dysfunction, which causes disruptions in neurovascular coupling, hypometabolism in neurons and oxidative stress [19–21]. However, the measurement of these biological factors was beyond the scope of this study. Future investigations will be necessary to support these connections between neural dynamics, immune health and vascular health in treating post-COVID syndrome and supporting brain health in general.

Importantly, after a 1-month (eight sessions lasting around 1-h each) adaptive task-switching training, intrinsic neural processing differences between our healthy and post-COVID old females were no longer significant. Previous studies showed that cognitive training in older individuals influences both cognitive function and structural/functional brain efficiency [126–130]. In our case, cognitive training seemed to modulate intrinsic neural dynamics for older women, thereby reducing differences between patients and controls. Even though we cannot rule out time effects entirely, the possibility that the post-COVID-syndrome-related neural changes resolved themselves as time went on is very low. Our post-COVID participants reported no significant improvement in their subjective symptoms during the adaptive cognitive training procedure. At a first glance, this lack of improvement in post-COVID symptoms may negate the importance of cognitive training in post-COVID patients. However, we did not expect that our cognitive training protocol with one cognitively demanding task lasting 8 h would improve post-COVID-related symptomology generally. Rather, we suggest that cognitive training supported less effortful task performance in post-COVID females through the attenuated increase of local information processing capacity, and consequently, they showed similar intrinsic neural dynamics underlying similar behavioural performance as compared to their healthy peers after the training. Therefore, our study supports the efficiency of cognitive training in improving neural and cognitive functioning in post-COVID patients and is a crucial first step towards developing more complex and possibly longer training interventions in the future. However, it is important to note that healthy old females made significantly faster responses compared to post-COVID old females post-training (but the two groups did not differ in hit rate, MC and SC after training). In our previous study [131], we found that even in healthy older adults (females), the adaptive task-switching training could modulate the age-related shift from more global to local information processing as revealed by the increased coarse scale entropy and lower frequency band power from pre- to post-training condition at specific electrodes. Therefore, we suggested that healthy older adults (females) gain lasting large-scale neural processing benefits and flexibility from adaptive cognitive training which can be detected in behavioural performance as well.

To summarize the modulatory effect of our adaptive task-switching training protocol, we found that post-COVID older males benefited the least. Although their behavioural performance improved, their neural processing effort remained higher. In contrast, post-COVID older females required less cognitive effort to achieve improved performance. Meanwhile, healthy older females demonstrated more flexible large-scale brain dynamics along with better performance. We specifically applied this cognitive training protocol because it relies on cognitive control functions and the distributed connectivity between prefrontal, frontoparietal and basal ganglia regions which are the most sensitive to the aging process. Since resting-state neural dynamics are connected to cognitive functioning and performance, this type of cognitive training is suitable to detect changes in intrinsic neural plasticity and reveal distributed training-related effects outside of the trained task. Based on our results, it is a possibility that with cognitive training, we can achieve distinct goals in specific populations depending on different levels of plasticity and other neural, psychological and biological factors [165–169]. While in patient groups we could detect more compensatory effects, in healthy groups, we can find more maintenance and gaining effects. However, future studies will need to elaborate on this aspect of training differences.

Our study sheds light on important yet less investigated aspects of post-COVID syndrome regarding the altered neural information processing capacity and adaptability, sex-related differences and also the promising modulatory effect of cognitive training in this condition specifically in females. More importantly, this line of inquiry could also be relevant in the future for investigating the neural underpinnings of cognitive features in other debilitating syndromes. Post-COVID syndrome shows great similarity with general post-viral syndrome [170–172], myalgic encephalomyelitis/chronic fatigue [14, 25, 173–177] and biological aging [41]. Since we know very little about these conditions and they are hard to diagnose, examining the growing number of post-COVID patients and the neural, immune and physiological background of this chronic condition could be greatly beneficial for understanding these chronic states, illnesses and non-pathological/pathological aging processes and finding diagnostic indicators for them.

Even though this study made crucial steps toward understanding the aetiology of post-COVID syndrome, the affected levels by the chronic state of COVID-19 infection and the individual sensitivity to it, it was evaluated in a more exploratory way since there was only scarce evidence in the literature regarding the post-COVID- and sex-related differences in neural dynamics and information processing. Additionally, our study has some limitations. Our healthy and mainly our post-COVID groups were highly intelligent and as mentioned before, they may have extensive cognitive reserve to compensate the age- and post-COVID-related effects [156–161]. Also, resting-state neural dynamics are connected to the efficiency of cognitive functioning [63–70]; thus, it would be crucial to study older adults with more diverse backgrounds and cognitive abilities in the future. Regarding the sex-related differences, we cannot rule out that the detected differences are general sex effects as opposed to specific post-COVID-mediated ones; thus, further studies will be needed to address this question. Albeit our study focused on how post-COVID syndrome affected intrinsic neural dynamics and we show a more pronounced shift to local neural information processing in older post-COVID patients compared to healthy ones, future research should comprehensively investigate this effect as a possible accelerated neural aging process or as a neural compensatory mechanism. It is crucial to include various age groups and both genders and larger sample sizes to understand the neural effects of and compensatory interventions for post-COVID syndrome. Also, it is possible that the aging mechanism suppressed the sex-related differences and sensitivity in post-COVID syndrome; thus, examining younger patients would be necessary in this regard as well.

In summary, our study revealed that the chronic phase of COVID-19 infection had a similar impact on the intrinsic neural processing for both females and males. However, our adaptive task-switching training paradigm modified local neural dynamics for females but not for males. Moreover, when comparing the brain dynamics of similarly aged healthy and post-COVID females prior to training, we detected increased local information processing capacity in patients, evidenced by increased finer timescale entropy (1–30 ms) and higher frequency band power (11–40 Hz). These results suggest that there is a more pronounced age-related shift from more global to local neural processing in post-COVID patients, potentially linked to the hypothesized accelerated aging process in post-COVID syndrome or to the neural compensatory mechanism for achieving similar performance. Notably, this difference disappeared with cognitive training, indicating the potential modulatory effect of cognitive interventions in post-COVID syndrome, particularly among females. These results also underscore the importance of separately considering males and females in medical treatment.

## Supplementary Information

Below is the link to the electronic supplementary material.

Supplementary file1 (DOCX 1320 KB)

## Data Availability

The datasets analysed during the current study are available in the G-Node GIN repository: https://gin.g-node.org/gaalzs/POST_COVID_REST.
