# Supplementary material for: Resting-state neural dynamics changes in older adults with post-COVID syndrome and the modulatory effect of cognitive training and sex
Source: GeroScience. 2024 Aug 29;47(1):1277–301. doi: 10.1007/s11357-024-01324-8 (PMC11872858; doi:10.1007/s11357-024-01324-8)
Supplement: Supplementary file 1 — Supplementary file1 (DOCX 1320 KB) [file 11357_2024_1324_MOESM1_ESM.docx]

**Supplementary Material**

For the manuscript submitted to GeroScience:

Resting-state neural dynamics changes in older adults with post-COVID syndrome and the modulatory effect of cognitive training and sex

Boglárka Nagy, Andrea B. Protzner, Balázs Czigler, Zsófia Anna Gaál

Corresponding author: Boglárka Nagy

Institute of Cognitive Neuroscience and Psychology, HUN-REN Research Centre for Natural Sciences, Budapest, Hungary

Email: [nagy.boglarka@ttk.hu](mailto:nagy.boglarka@ttk.hu)

**S3. Results**

**S3.3. Comparison of the three experimental groups regarding the effect of post-COVID syndrome, cognitive training, and sex on intrinsic neural dynamics**

In the main analyses of this study, we explored separately the sex differences and the healthy/patient comparison among older females, examining changes in neural dynamics associated with post-COVID syndrome and the effects of cognitive training interventions. However, comparing the resting-state neural information processing differences in our three older training groups (healthy old-female, post-COVID old-female, post-COVID old-male) could support and supplement our main results. Thus, we ran three-group task PLS analyses on resting-state MSE and SPD data for detecting the neural changes during cognitive training and if the intrinsic brain dynamics of our experimental groups significantly differed before and after the cognitive training.

Regarding multiscale entropy, we found different pattern changes with cognitive training in the experimental groups through the significant latent variable (LV1, singular value = 1.17, p = 0.004, Figure S1, Panel A). Namely, post-COVID old-females showed decreased, while healthy old-females showed increased entropy, with a more widespread manner in the 1-30 ms timescales and more sparsely in coarser timescales (30-50 ms). However, the training-related change in resting-state neural complexity was not significant in the post-COVID old-male group (revealed by the confidence intervals around the MSE design saliences which are crossing 0 in this group) which implies that this intervention method was less effective in this group. In connection with the effect of post-COVID syndrome on intrinsic neural complexity, both older female and male post-COVID participants showed general increase in fine and middle scale entropy (1-30 ms) compared to healthy older females before training which was revealed by the one significant latent variable (LV1, singular value = 1.77, p = 0.002, Figure S1, Panel B). However, we found no differences between the intrinsic neural processes of our experimental groups after cognitive training since we did not detect any significant latent variable in the post-training comparison (Figure S1, Panel C).

Applying a similar approach to the complementary spectral power density analysis, we also detected different pattern changes with cognitive training across the experimental groups through the significant latent variable (LV1, singular value = 94.12, p = 0.002, Figure S2, Panel A). Both female and male post-COVID patients showed decreased power in higher frequencies mainly in beta and lower gamma bands and increased power in delta frequency band during cognitive training, while healthy older females showed the opposite pattern. Examining the effect of post-COVID syndrome on relative oscillatory power in older adults, we found that both sexes with post-COVID showed increased power in higher frequency bands from upper alpha to beta and lower gamma, and decreased power in delta band compared to healthy older females before training. This was revealed by the one significant latent variable in the pre-training analysis (LV1, singular value = 155.38, p < 0.002, Figure S2, Panel B). However, these differences disappeared with cognitive training as shown by the post-training SPD data where no significant latent variable, thus no significant difference was found between our experimental groups (Figure S2, Panel C).

Altogether, these results further supported our main findings regarding the increased local information processing capacity in older post-COVID patients, indicative a pronounced shift from more global to local neural dynamics compared to healthy peers. Moreover, our task-switching training protocol successfully modulated the post-COVID-related alterations in intrinsic brain dynamics, with greater efficacy observed in females compared to males.

**
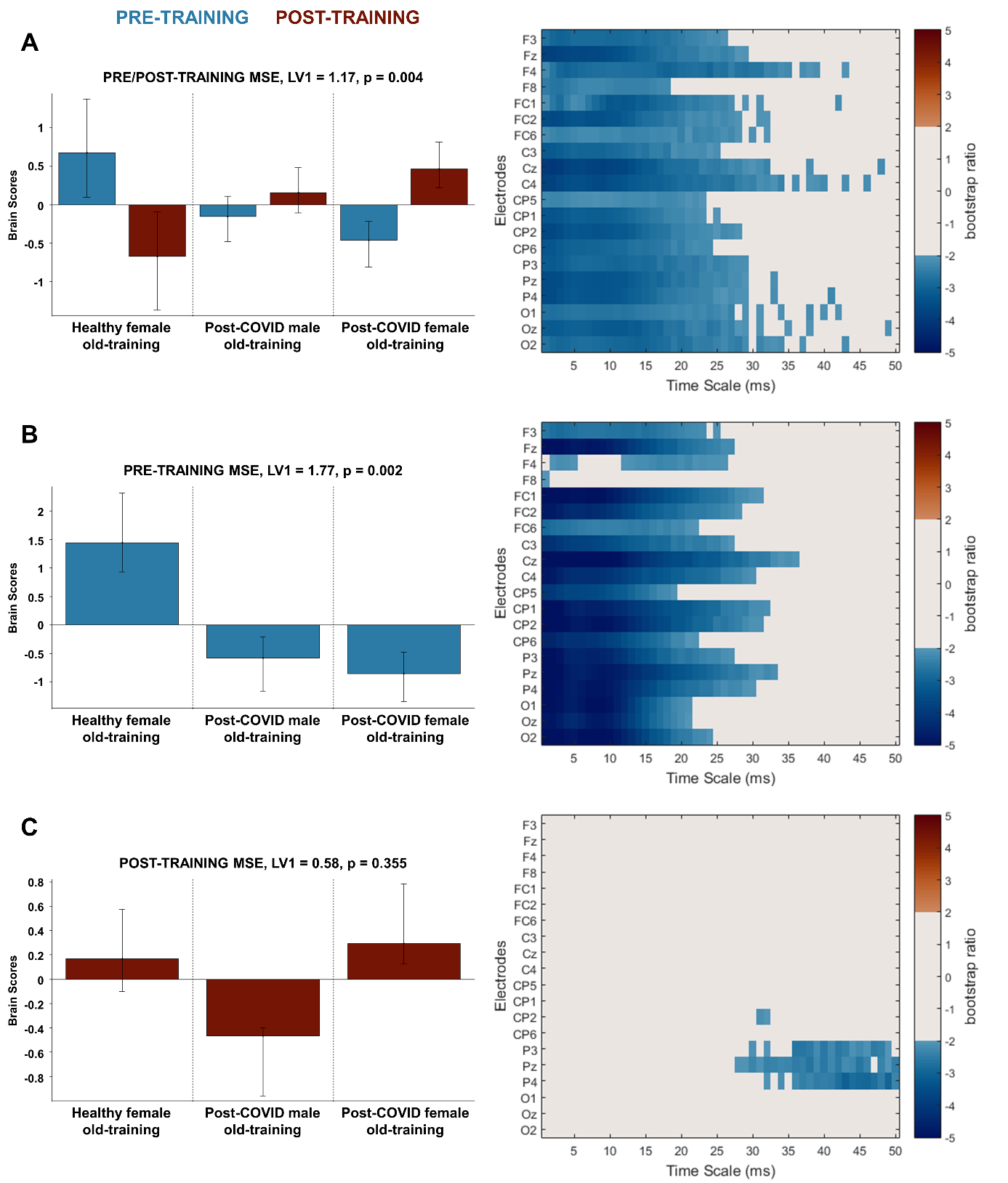
**

**Figure S1.** PLS analysis results for the effect of post-COVID syndrome and cognitive training on resting-state MSE in all of our experimental groups. **Panel A** shows the significant contrast and interaction between experimental groups (healthy old-female/post-COVID old-male/post-COVID old-female) and training conditions (pre-training/post-training); **Panel B** shows the significant contrast between the three experimental groups before cognitive training; and **Panel C** shows the lack of significant contrast between the three groups after cognitive training. On the left side of every panel the bar graph depicts the contrast that was significantly expressed across electrodes and timescales as determined by permutation tests, with error bars denoting 95% confidence intervals. On the right side of every panel the bootstrap ratio map illustrates the electrodes and timescales at which the contrast displayed in the bar graphs was significant. Values represent the ratio of the individual electrode weights and the bootstrap-derived standard error (thresholded at 2.0 which corresponds approximately to p < 0.05) where positive values are plotted in warm colours and negative values in cool colours. In **Panel A**, negative values indicate timescales and electrodes showing increases in healthy old-female group and decreases in post-COVID groups (which was significant in old female patients but not in old males) from pre- to post-training in resting-state MSE. In **Panel B**, negative values indicate timescales and electrodes showing increased resting-state MSE in both female and male post-COVID groups compared to the healthy old-female group in the pre-training condition. In **Panel C**, the contrast between healthy and post-COVID groups in resting-state MSE is not significant post-training.

**
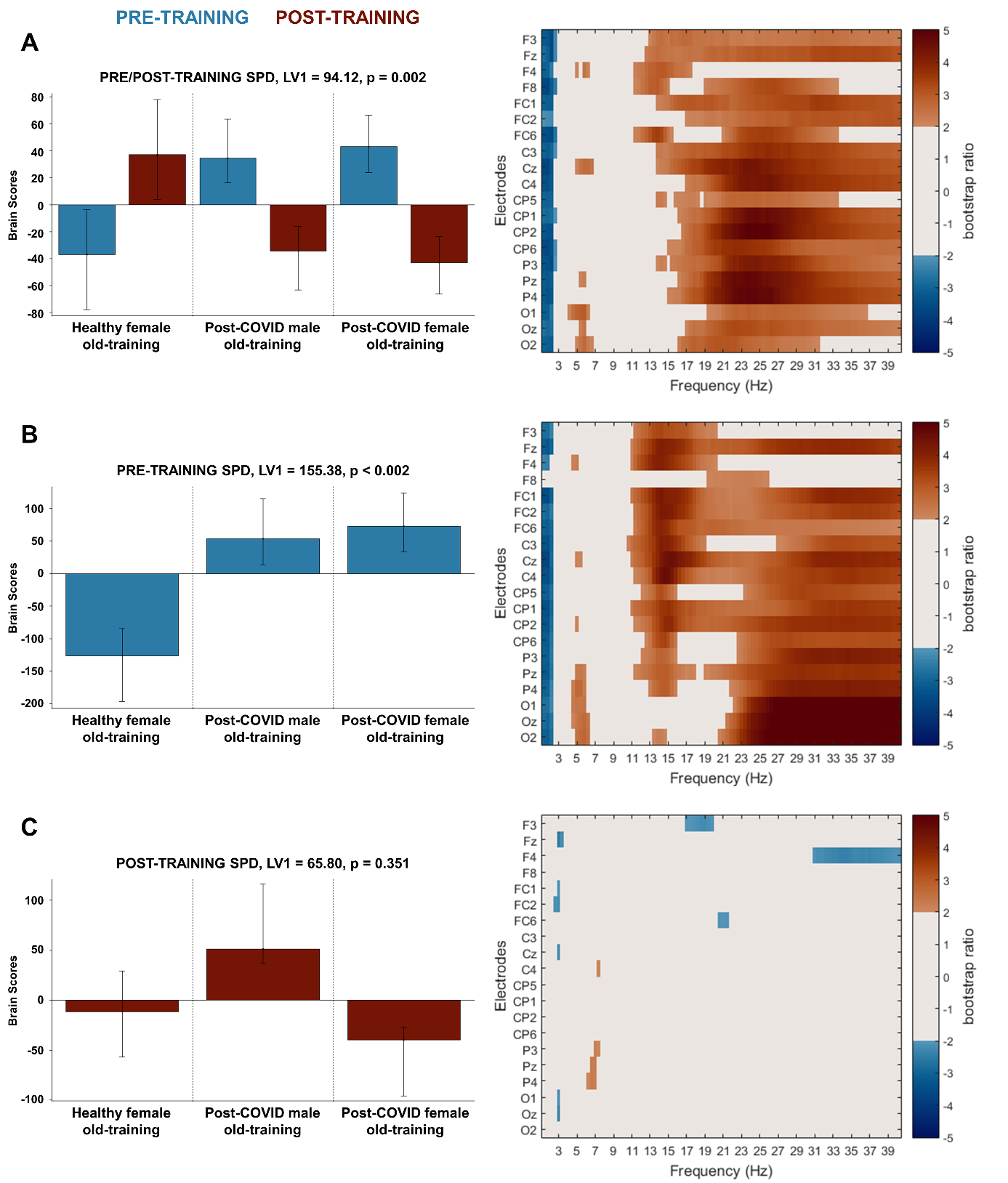
**

**Figure S2.** PLS analysis results for the effect of post-COVID syndrome and cognitive training on resting-state SPD in all of our experimental groups. **Panel A** shows the significant contrast and interaction between experimental groups (healthy old-female/post-COVID old-male/post-COVID old-female) and training conditions (pre-training/post-training); **Panel B** shows the significant contrast between the three experimental groups before cognitive training; and **Panel C** shows the lack of significant contrast between the three groups after cognitive training. On the left side of every panel the bar graph depicts the contrast that was significantly expressed across electrodes and frequencies as determined by permutation tests, with error bars denoting 95% confidence intervals. On the right side of every panel the bootstrap ratio map illustrates the electrodes and frequencies at which the contrast displayed in the bar graphs was significant. Values represent the ratio of the individual electrode weights and the bootstrap-derived standard error (thresholded at 2.0 which corresponds approximately to p < 0.05) where positive values are plotted in warm colours and negative values in cool colours. In **Panel A**, positive values indicate frequencies and electrodes showing increases in the healthy old-female group and decreases in post-COVID old-female and old-male groups from pre- to post-training in resting-state SPD; while negative values indicate frequencies and electrodes showing decreases in the the healthy old-female group and increases in post-COVID groups from pre- to post-training in resting-state SPD. In **Panel B**, positive values indicate frequencies and electrodes showing increased resting-state SPD in both female and male post-COVID groups compared to the healthy old-female group in pre-training condition; while negative values indicate frequencies and electrodes showing decreased resting-state SPD in the post-COVID groups compared to the healthy old-female group in pre-training condition. In **Panel C**, the contrast between the healthy and post-COVID groups in resting-state SPD is not significant post-training.
